# Influence of antiresorptive/antiangiogenic therapy on the extension of experimentally induced peri-implantitis lesions

**DOI:** 10.1007/s00784-023-04904-8

**Published:** 2023-02-17

**Authors:** Frank Schwarz, Kathrin Becker, Fanya Lukman, Katharina Melissa Müller, Victoria Sarabhai, Nicole Rauch, Robert Kerberger, Ausra Ramanauskaite, Robert Sader, Karina Obreja

**Affiliations:** 1 Department of Oral Surgery and Implantology, Goethe University, Frankfurt am Main, Germany; 2 Department of Orthodontics, Universitätsklinikum Düsseldorf, Düsseldorf, Germany; 3 Department of Oral Surgery, Universitätsklinikum Düsseldorf, Düsseldorf, Germany; 4 Department for Oral, Cranio-Maxillofacial and Facial Plastic Surgery, Medical Center of the Goethe University, Frankfurt am Main, Germany

**Keywords:** Animal experiment, Peri-implantitis, Antiresorptive therapy, Antiangiogenic therapy, Histological technique

## Abstract

**Objectives:**

To investigate the extension of experimentally induced peri-implantitis lesions under various antiresorptive and antiangiogenic medications.

**Material and methods:**

Fourty-eight albino rats had randomly received the following medications (dual application, *n* = 8 each): (1) amino-bisphosphonate (zoledronate) (Zo), (2) RANKL inhibitor (denosumab) (De), (3) antiangiogenic (bevacizumab) (Be), (4) Zo+Be, (5) De+Be, or (6) no medication (Co). Ligature- and lipopolysaccharide-induced peri-implantitis lesions were established at 2 maxillary implants over a period of 16 weeks. Histological (e.g., apical extension and surface area of the inflammatory cell infiltrate—aICT, ICT; defect length; defect width; CD68 positive cells) and bone micromorphometric (μCT) outcomes were assessed. The animal was defined as a statistical unit.

**Results:**

A total of *n* = 38 animals (Zo = 6, De = 6, Be = 8, Zo + Be = 6, De + Be = 5, Co = 7) were analyzed. ICT’s were commonly marked by a positive CD68 antigen reactivity. Comparable median aICT (lowest—Zo: 0.53 mm; highest—Be: 1.22 mm), ICT (lowest—De + Be: 0.00 mm^2^; highest—Co: 0.49 mm^2^), defect length (lowest—Zo: 0.90 mm; highest—Co: 1.93 mm) and defect width (lowest—De+Be: 1.27 mm; highest—Be: 1.80 mm) values were noted in all test and control groups. Within an inner (diameter: 0.8 mm) cylindric volume of interest, the bone microstructure did not significantly differ between groups.

**Conclusions:**

The present analysis did not reveal any marked effects of various antiresorptive/ antiangiogenic medications on the extension of experimentally induced peri-implantitis lesions.

**Clinical relevance:**

The extension of peri-implantitis lesions may not be facilitated by the antiresorptive and antiangiogenic medications investigated.

## Introduction

Peri-implantitis lesions are plaque-biofilm related inflammatory conditions affecting all tissues surrounding dental implants in function, thus requiring antiinfective therapy [1–3].

Recent clinical case reports suggest a potential association between peri-implantitis lesions and a medication-related osteonecrosis of the jaw (MRONJ) [4–6]. MRONJ has been linked with various antiresorptive drugs, such as bisphosphonates (e.g. amino-bisphosphonate zoledronic-acid—Za) and inhibitors of receptor activator of NF-κB ligand—RANKL), but also antiangiogenic medications [7–10]. At the time being, it is unknown whether peri-implantitis lesions may in fact trigger the occurrence of MRONJ. Conversely, it is also unknown whether the pro-inflammatory effects noted e.g. for Zo [11–13] or the potential influence of RANKL inhibitors on the host inflammatory response (i.e., T cells, B cells, or monocyte—macrophages) [14, 15] may influence the pathogenesis of peri-implantitis.

A systematic review and meta-analysis has associated MRONJ with an increased prevalence of periodontitis (risk ratio of 2.75) [16]. While periodontitis and peri-implantitis do share some similarities [17–19], the inflammatory lesions at peri-implantitis sites were noted to be larger in size and characterized by larger proportions of polymorphonuclear leukocytes and macrophages than those assessed at periodontitis sites [20, 21]. Accordingly, the host inflammatory response and subsequently the progression of peri-implantitis lesions may be further triggered by the pro-inflammatory effects noted for various antiresorptive/ antiangiogenic drugs.

Therefore, the aim of the present study was to investigate the extension of experimentally induced peri-implantitis lesions under antiresorptive and antiangiogenic medications in a rodent model. It is hypothesized that various types and common combinations of antiresorptive/antiangiogenic drugs promote the histological extension of the inflammatory lesions when compared with an untreated (i.e., without antiresorptive/antiangiogenic medication) control group.

## Material and methods

### Animals

Forty-eight albino rats of the Wistar strain (age: 6 months, mean weight 0.476 ± 0.5 kg; Janvier Labs, Sulzfeld, Germany) obtained from a certified breeder were used in the study. All animals were housed in appropriately dimensioned cages under standard conditions of temperature in a light-controlled environment and were provided water and special diet ad libitum. The study protocol considered the 3R (replace, reduce, refine) guidelines for animal experimentation and was approved by the appropriate local authority (Regierungspräsidium Darmstadt, Germany). The following reporting adhered to the ARRIVE Guidelines 2.0 [22].

### Study design and surgical procedures

**Fig. 1:**
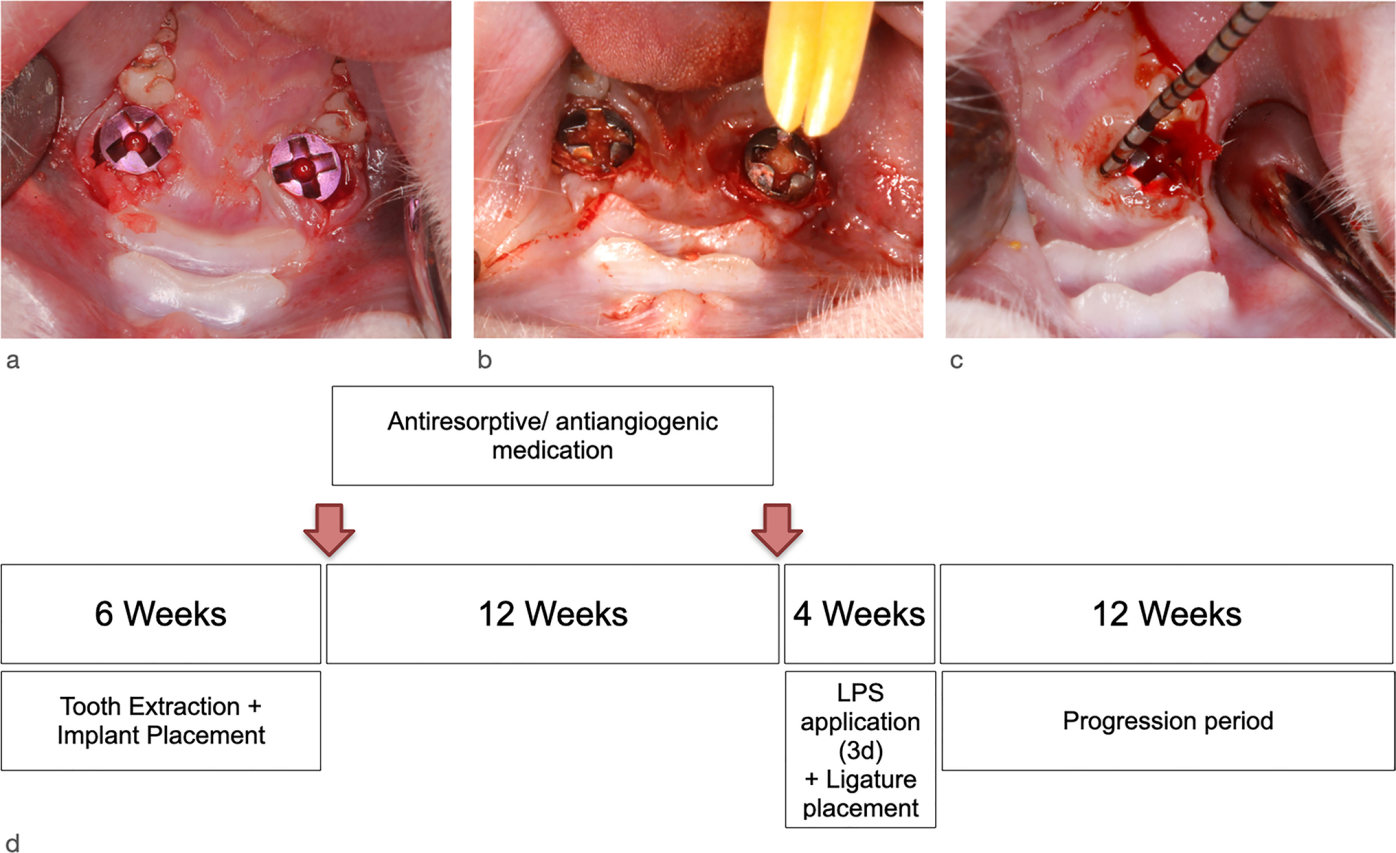
Experimentally induced peri-implantitis lesions. **a** Situation following immediate implant placement bilaterally in the region of the maxillary first molars. All sites were left to heal for 6 weeks, after which the first drug administration was provided to the animals of the test groups. Drug administration was repeated at 12 weeks. **b** Ligature placement (polyester (6-0) in a submucosal position. This was associated with a single intraperitoneal and repeated topical LPS application for 3 days. Ligatures were removed after 4 weeks. **c** Clinical signs of inflammation at the end of the progression period at 12 weeks as indicated by mucosal hyperplasia, bleeding on probing, and suppuration. **d** Study outline and timing of the experimental phases

Following the extraction of both maxillary first molars, smooth-surfaced titanium mini-implants (Ustomed® Micro-Screws, Cross, ⌀ 1.2 mm, shortened to 3 mm) were immediately inserted [23] at respective sites and left to heal for 6 weeks. Subsequently, the animals had randomly (block randomization, Randlist, DatInf GmbH, Tübingen, Germany) received the following commonly applied antiresorptive/ antiangiogenic medications, including *n* = 8 animals each: (1) amino-bisphosphonate (Zoledronate 5mg/ kg intravenous, Ribometa® 4mg/5ml, Hikma Pharma, Gräfelfing, Germany) (Zo); (2) RANKL inhibitor (Denosumab 60mg/kg subcutaneous, Prolia®, Amgen, Munich, Germany) (De); (3) antiangiogenic medication (Bevacizumab 5mg/kg intravenous, Avastin® 400 mg/16 ml, Roche Pharma, Grenzach-Wyhlen, Germany) (Be); (4) Zo + Be; (5) De + Be; or (6) no medication serving as the control group (Co). Drug administration was repeated at 12 weeks. Afterwards, peri-implantitis lesions were induced by an established and validated procedure [24]. This included an intraperitoneal booster lipopolysaccharide (lipopolysaccharide *Escherichia coli* O111:B4, EMD Millipore, Merck, Darmstadt, Germany) injection along with daily topical injections in the peri-implant sulcus at each implant site for 3 days. In a next step, miniature polyester ligatures (Dagrofil 6-0, B. Braun, Melsungen, Germany) were placed in a submarginal position around both implants in each animal for 4 weeks. In brief, ligatures were forced into a position directly apical of the mucosal margin. The resulting “pocket” facilitated the establishment of a submucosal microflora. This was followed by a progression period of 12 weeks (Fig. 1). During the progression period, a total of *n* = 44 implants (Zo = 7, De = 7, Be = 6, Zo + Be = 6, Co = 9) were lost. Accordingly, a total of *n* = 38 animals (Zo = 6, De = 6, Be = 8, Zo + Be = 6, De + Be = 5, Co = 7) exhibiting *n* = 52 implants (Zo = 9, De = 9, Be = 10, Zo + Be = 10, De + Be = 7, Co = 7) were available for the histomorphometrical analysis.

All surgical procedures were performed by experienced surgeons (F.S., K.O., A.R.).

### Anesthesia protocol

For each surgical intervention, the animals were anesthetized by intraperitoneal injection of 7.5 mg/kg ketamine (Ketanest®, Pfizer Pharma GmbH, Karlsruhe, Germany) and 5 mg/kg xylazine (Rompun®, Bayer HealthCare, Leverkusen, Germany). For postoperative analgesia, 4.5 mg/kg carprofene was administered subcutaneously immediately after surgery, as well as 1, 2, and 3 days postoperatively.

### Histological and immunohistochemical processing

The animals were euthanized with an overdose of pentobarbitone at 100 mg/kg. All specimens were fixed in 10% neutral buffered formalin solution for 10 days.

Tissue blocks were decalcified using ultrasound supported water bath and Ethylenediaminetetraacetic for 12 weeks. Prior to processing and embedding in paraffin, the implants were carefully removed by counterclockwise unscrewing. Two most central sections of each block were cut in the horizontal plane with the micrometer set at 3–4 μm. One section per block was stained with hematoxylin and eosin (HE). The second section was used for immunohistochemical staining for cluster of differentiation 68 (CD68). After deparaffinization and rehydration of the tissue sections, antigen unmasking was performed by heating for 15 min in target retrieval solution (DakoCytomation, Hamburg, Germany). A primary mouse anti-rat monoclonal antibody was used to stain CD68 (dilution 1:500, DKFZp686M18236, GP110, Macrosialin, Scavenger Rece, Biomol, Hamburg) for 30 min. The slides were washed in phosphate-buffered saline and incubated with a secondary biotinylated anti-mouse antibody (1:60 dilution, DakoCytomation) for 1 h at room temperature. The presence of antibody-antigen complexes was visualized using a streptavidin-peroxidase solution (1:300 dilution, DakoCytomation) with AEC as the chromogen (DakoCytomation). For negative controls, the primary antibody was replaced with non-immune serum.

### Histological and histomorphometrical analysis

Digital images (BX53, Olympus, Hamburg, Germany) were obtained from each specimen and evaluated using a software program (cellSens, Olympus).

**Fig. 2:**
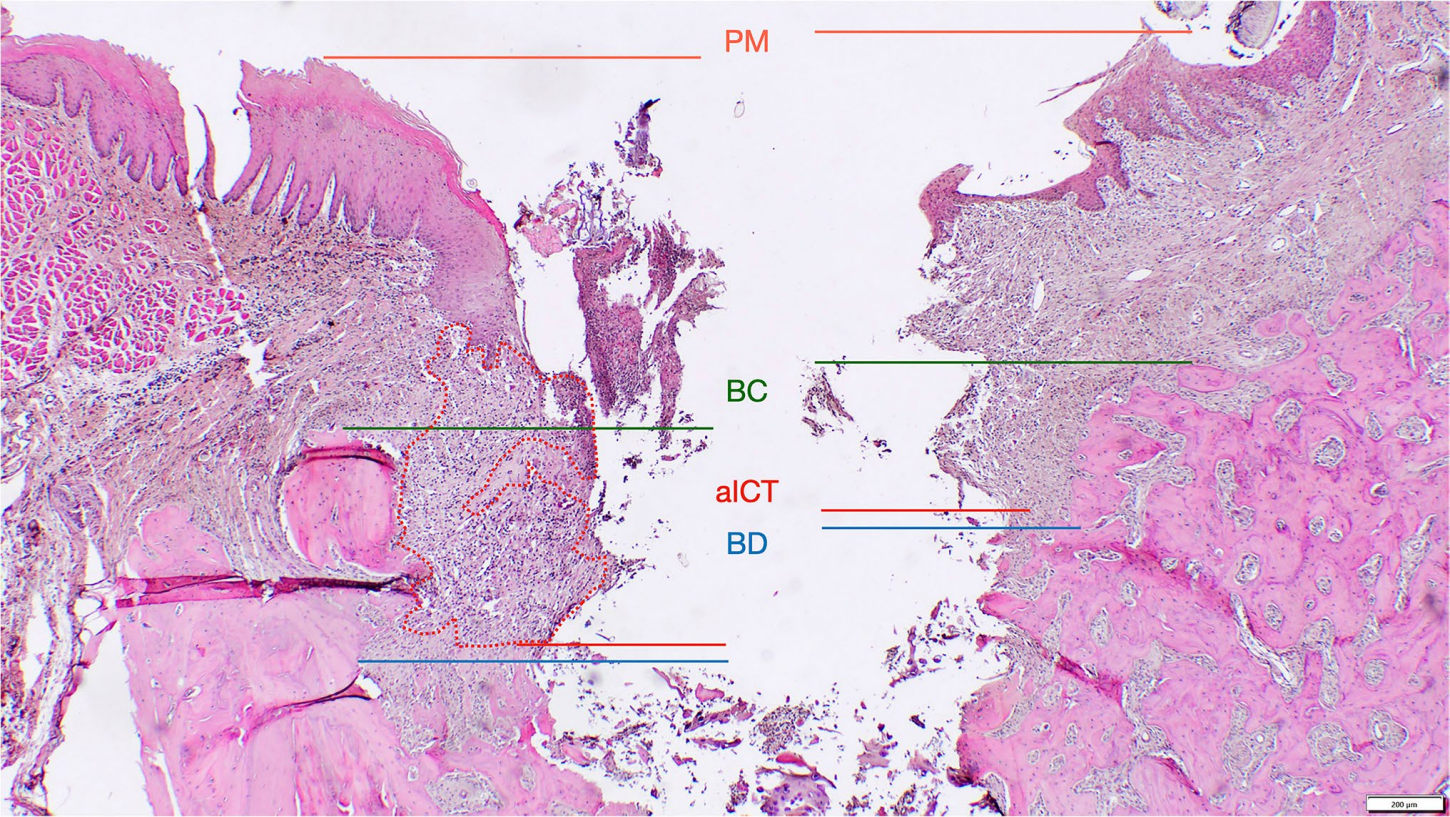
Landmarks and outcomes defined for the histomorphometrical analysis—the mucosal margin (PM), the apical extension (aICT) and surface area of the inflammatory cell infiltrate (ICT), the bone crest (BC), the defect bottom (BD). Defect length (DL) was measured from BC to BD and defect width (DW) was measured from the vestibular to the oral BC

The following landmarks were identified in the histological sections at each experimental site and at both vestibular and oral aspects (Fig. 2) [25, 26]: the mucosal margin (PM), the bone crest (BC), and the defect bottom (BD).

Linear measurements were made by drawing a vertical line, following the long axis of the implant bed, from PM to the apical extension of the inflammatory cell infiltrate (aICT), BC to BD (i.e., defect length). The horizontal defect width was measured from the vestibular to the oral BC. The surface area (mm^2^) of the ICT was assessed using an implemented edge detection tool. All measurements were performed by two previously calibrated examiners (F.L., K.M.). Calibration was accepted when repeated measurements of *n* = 5 different sections were similar at > 95% level.

### Image processing and analysis of the bone micromorphometry

**Fig. 3:**
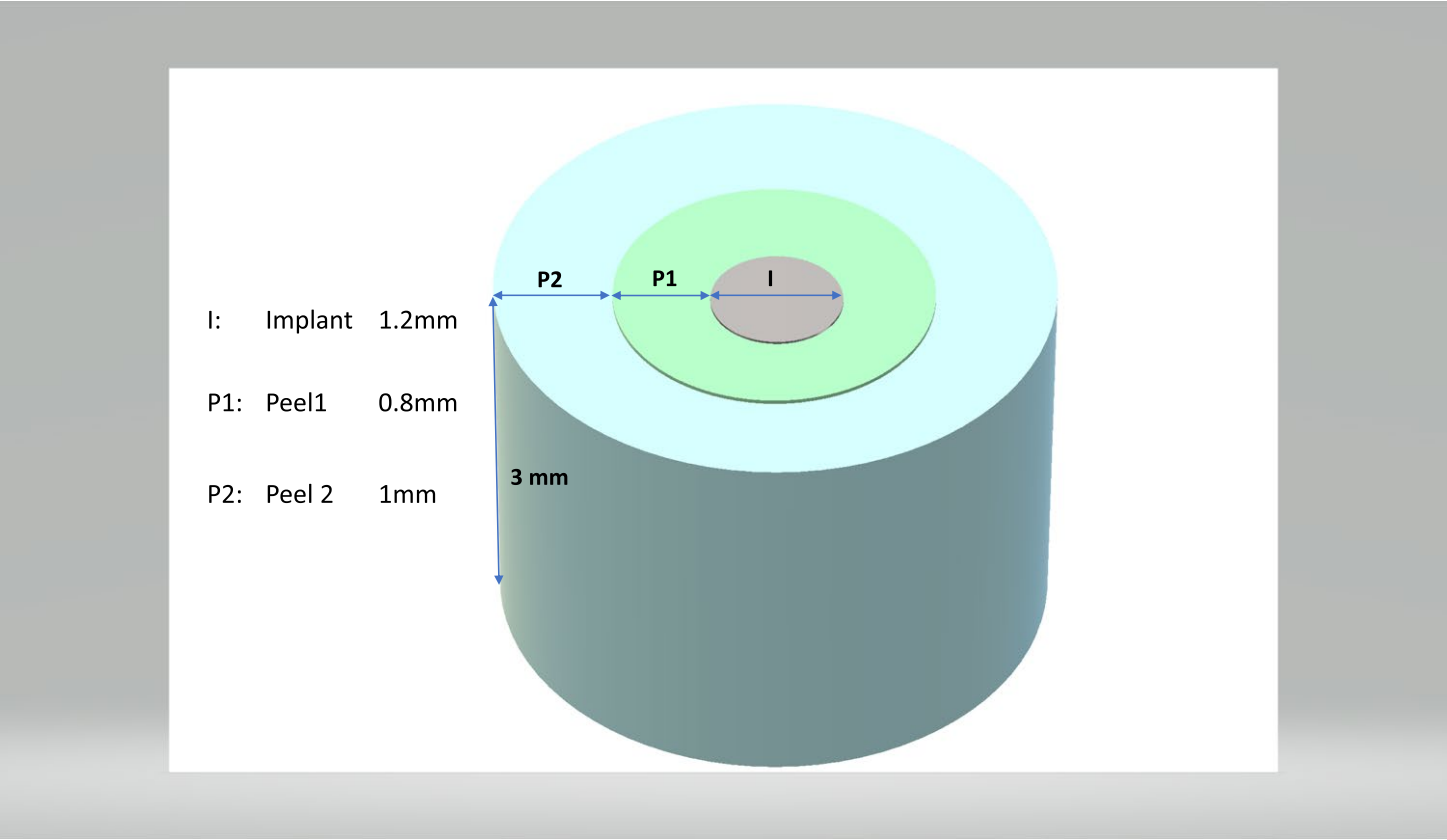
Representation of the volumes of interest (VOI) used for the measurement. Notation: I: Implant (diameter: 1.2 mm, height: 3.0 mm), P1: peel 1 (inner VOI around I, width 0.8 mm, height: 3.0 mm), P2: peel 2 (outer VOI around I, width: 1.0 mm, height: 3.0 mm)

**Fig. 4:**
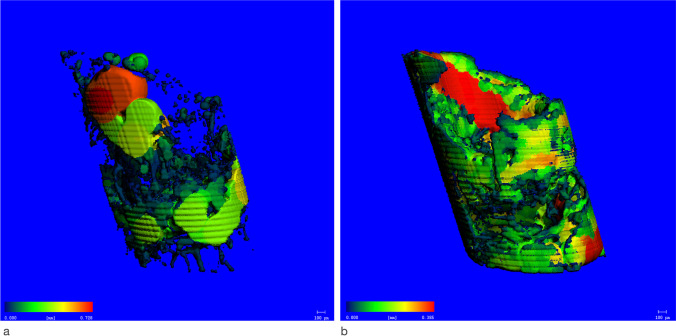
Colour-coded representation of bone micromorphometric outcomes. Regions of high thickness are marked in red, whereas low thickness is marked in blue. **a** Tb.Sp (Zo + Be group). **b** Tb.Th (Zo + Be group)

Prior to decalcification, specimens were scanned using a μCT device (Viva CT 80, Scanco Medical AG, Brüttisellen, Switzerland) at 70 kVp, 114 μA, and 786 ms of integration time. The scans were reconstructed to a nominal voxel size of 16.1 μm and analyzed using the dedicated evaluation software provided by the manufacturer (Scanco Medical). The scans were aligned to the original implant axis and occlusion plane. Then, around the former implant (diameter: 1.2 mm, height 3 mm), an inner (diameter: 0.8 mm, peel 1) and outer (diameter: 1.0 mm, peel 2) cylindric volume of interest (VOI) was identified by a calibrated observer (V.S.) (Fig. 3). Within these VOIs, the following indices were evaluated: bone volume per total volume (BV/TV) representing the bone quantity within the selected VOI; the Bone Mineral Density (BMD) expressed as the mean amount of hydroxyapatite within tissue segmented as bone in the VOI (mgHA/mm^3^); the trabecular thickness (Tb.Th) indicating the width of bone structures (mm) (Fig. 4a); the trabecular spacing (Tb.Sp) which represents the width of the uncalcified regions (mm) (Fig. 4b), and the specific bone surface (BS/BV) (mm^2^/mm^3^) representing the ratio of surface area to the bone volume within the VOI.

### Sample size calculation

For the power analysis, a standard normal distribution was assumed. The probability of a type I error was set at .05. The effect size d (7.71) was calculated based on the means and standard deviations of the mean progressive bone loss observed in a previous animal study employing a similar defect model [27]. The margin for the primary outcome defect length was set at 0.5 mm. In order to achieve 95% power, a sample size of 6 + 2 (i.e., to account for a drop-out rate of about 30%) animals per group was calculated (G * Power 3.1).

### Statistical analysis

The statistical analysis of the data sets was performed using the software program R [28] defining the animal as a statistical unit. The R package *ggplot2* [29] was used to create boxplots for descriptive analyses. The R package *lme4* [30] was used to perform linear mixed effects analyses to assess differences in histological and bone micromophometric parameters between groups. As fixed effects, the respective parameters were entered into the model. For random effects, intercepts for the animals were defined. Visual inspection of residual plots did not reveal any obvious deviations from homoscedasticity or normality. *P*-values were obtained by likelihood ratio tests of the full model with the effect in question against the model without the effect in question. Results were found to be significant at *P* < 0.05. Due to the limited final sample size of De + Be-treated animals (*n* = 5), this group was excluded from the statistical analysis.

## Results

### Histomorphometrical analysis

**Fig. 5:**
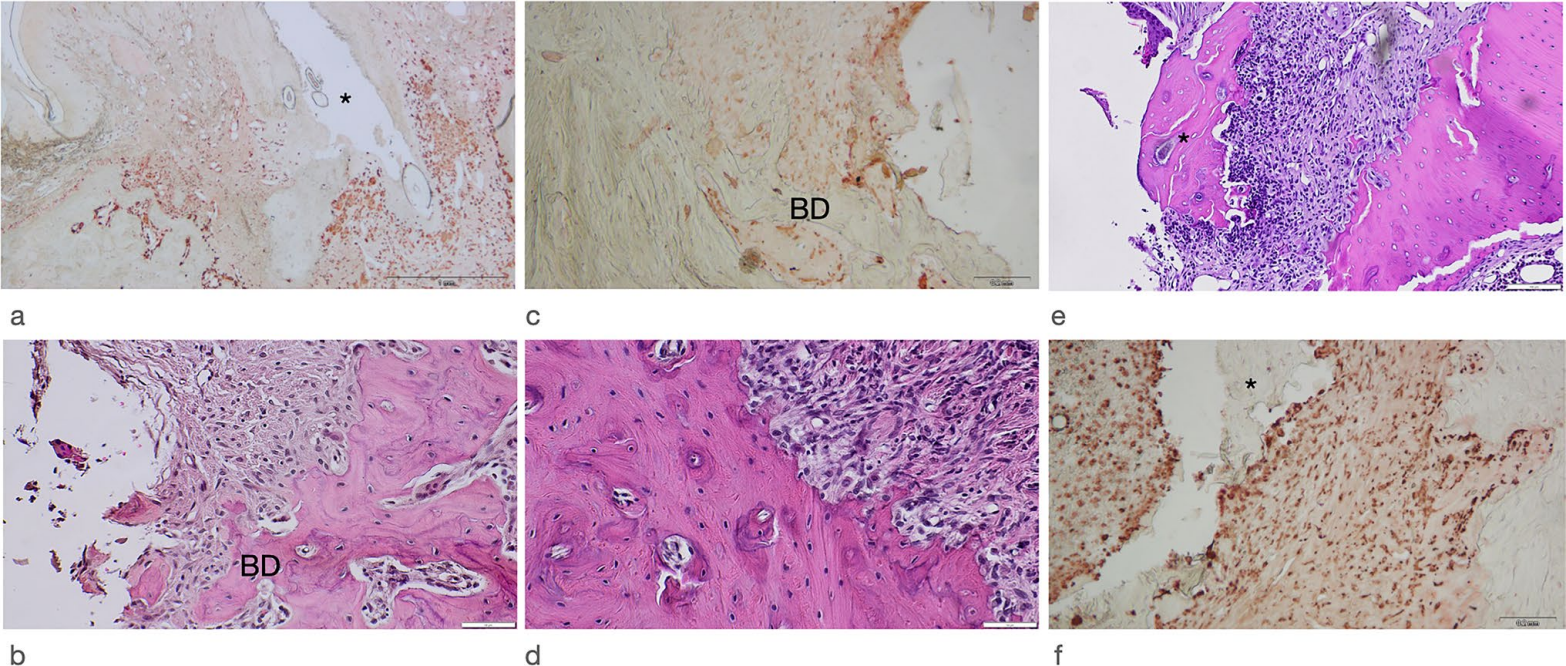
Representative histological and immunohistochemical views in different groups. **a** All groups revealed a chronic-type ICT with a marked CD68 antigen reactivity (asterisk = former implant position; Be group). **b** Apical extension of ICT in close proximity to BD (Co group, original magnification ×400). **c** Increased density of CD68 positive cells lining the frequently noted resorption lacunae at BD (Co group). **d** Resorption lacunae and histological signs of bone remodeling. A reduced number of osteocytes or the presence of empty lacunae was commonly not noted (Zo group). **e** Bone sequestrations (asterisk) were occasionally observed in all test and control groups (Zo group). **f** Increased CD68 reactivity adjacent to the bony sequestrum (asterisk) (Zo group)

The mean aICT, ICT, defect width, and defect length values in different groups are summarized in Table 1. Representative histological and immunohistochemical views of the respective defect areas are depicted in Fig. 5.

**Table 1:** Histomorphometrical analysis (mean ± SD; 95% CI) of aICT (mm), ICT (mm^2^), defect length (DL) (mm), and defect width (DW) (mm) values in different groups are reported on animal level (*n* = 38 animals)

Group		aICT	ICT	DL	DW
Zo (*n* = 6)		0.61 ± 0.46	0.13 ± 0.20	1.10 ± 0.50	1.51 ± 0.41
	*95% CI*	0.12; 1.10	− 0.07; 0.34	0.57; 1.64	1.07; 1.94
De (*n* = 6)		1.00 ± 0.51	0.25 ± 0.22	1.22 ± 0.50	1.64 ± 0.59
	*95% CI*	0.46; 1.54	0.02; 0.49	0.70; 1.75	1.02; 2.26
Be (*n* = 8)		1.05 ± 0.71	0.27 ± 0.33	1.34 ± 0.68	1.74 ± 0.55
	*95% CI*	0.45; 1.64	0.00; 0.55	0.77; 1.92	1.27; 2.19
Zo+Be (*n* = 6)		0.77 ± 0.60	0.12 ± 0.15	1.41 ± 0.71	1.71 ± 0.49
	*95% CI*	0.14; 1.41	− 0.03; 0.28	0.66; 2.16	1.19; 2.23
De+Be (*n* = 5)*		0.85 ± 1.00	0.01 ± 0.02	1.30 ± 0.55	1.42 ± 0.58
	*95% CI*	− 0.39; 2.09	− 0.01; 0.03	0.60; 1.99	0.70; 2.15
Co (*n* = 7)		1.39 ± 1.25	0.56 ± 0.60	1.69 ± 0.83	1.83 ± 0.54
	*95% CI*	0.22; 2.55	0.01; 1.12	0.91; 2.46	1.32; 2.33
*P*-values		0.574	0.486	0.500	0.579

*Excluded from the statistical analysis due to a limited final number of animals

All groups investigated revealed an established subepithelial mixed, chronic-type inflammatory cell infiltrate, which was clearly marked by a positive CD68 antigen reactivity (Fig. 5a). The median aICT values amounted to 0.76 mm in the Co group. These values were similar in the Zo + Be (median: 0.72 mm), Zo, and De + Be groups, amounting to 0.53 and 0.66 mm, respectively. In contrast, median aICT values were higher in both Be and DE groups, amounting to 1.22 and 1.02 mm, respectively. The differences among groups did, however, not reach statistical significance (Table 1).

The resulting median ICT values amounted to 0.49 mm^2^ in the Co group and were commonly higher than those noted in all test groups investigated. In a descending order, median ICT values in the De, Be, Zo+Be, Zo, and De+Be groups were 0.28 mm^2^, 0.20 mm^2^, 0.07 mm^2^, 0.03 mm^2^, and 0.00 mm^2^, respectively (Table 1). The frequency and distribution of CD68-positive cells within the ICT did not markedly differ between tests and control groups. In all specimens investigated, the ICT facing the adjacent alveolar bone was associated with the presence of resorption lacunae and a more intense CD68 antigen reactivity.

The resulting intraosseous defect compartments were clearly definable (Fig. 5b–d). In particular, the median defect length amounted to 1.93 mm in the Co group. In a descending order, these values were 1.41 mm, 1.40 mm, 1.29 mm, 1.12 mm, and 0.90 mm in the Zo + Be, De + Be, De, Be, and Zo groups, respectively. The statistical analysis did not reveal any significant differences between groups (Table 1). Some specimens of all test and control groups revealed isolated sequestered bone fragments, which were demarcated by CD68-positive cells located in multiple resorption lacunae. Histological signs of an osteonecrosis zone (i.e. reduced number of osteocytes and presence of empty lacunae) were commonly not observed (Fig. 5e and f).

Median defect width amounted to 1.71 mm in the control group, with comparable values noted in all test groups investigated. In particular, median defect width was 1.80 mm, 1.65 mm, 1.58 mm 1.55 mm, and 1.27 mm in the Be, Zo+Be, De, Zo, and De+Be groups, without revealing any significant differences between groups (Table 1).

A premature implant loss noted during the progression period was associated with the lowest residual mean defect width values in the Co group, amounting to 0.87 ± 0.73 mm (median: 0.85 mm). The corresponding values were 1.20 ± 0.75 mm (median: 1.08 mm) in the Zo + Be-, 1.20±0.41 mm (median: 1.08 mm) in the De, 1.10 ± 0.32 mm (median: 1.17 mm) in the Be, and 0.93 ± 0.44 mm (median: 1.00 mm) in the Zo group, respectively.

### Volumetric analysis—bone micromorphometry

Due to a temporary unavailability of the μCT device, only scans of a total of *n* = 35 animals (Zo = 4, De = 6, Be = 8, Zo + Be = 6, De + Be = 5, Co = 6) exhibiting *n* = 46 implants were available for the volumetric analysis.

**Fig. 6:**
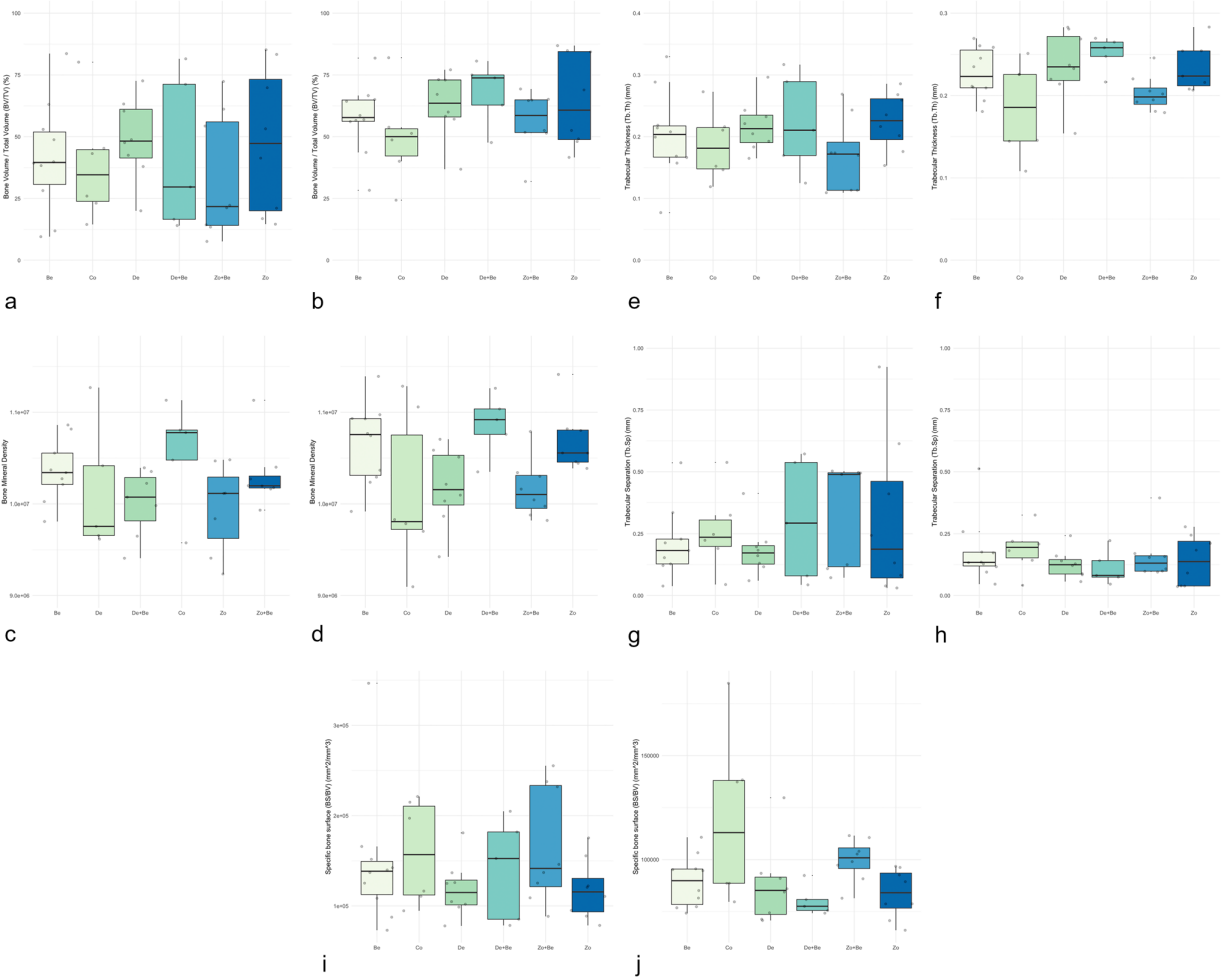
Boxplots depicting bone micromorphometric outcomes in test and control groups. **a** BV/TV (peel 1). **b** BV/TV (peel 2). **c** BMD (peel 1). **d** BMD (peel 2). **e** Tb.Th (peel 1). **f** Tb.Th (peel 2). **g** Tb.Sp (peel 1). **h** Tb.Sp (peel 2). **i** BS/BV (peel 1). **j** BS/BV (peel 2)

Boxplots depicting BV/TV, BMD, Tb.Th, Tb.Sp and BS/BV values in different groups in peels 1 and 2 are presented in Figure 6.

A huge variability in BV/TV values was noted among the test and control groups. In peel 1, the lowest values were seen in the De + Be and Zo + Be groups, whereas in peel 2, the lowest values were seen in the Co group. However, the statistical analysis failed to reveal any significant differences in peel 1 (*P* = 0.808) and peel 2 (*P* = 0.321) between tests and control groups (Fig. 6a and b).

The BMD values were comparable among test groups within peel 1 (Fig. 6c and d). The Tb.Th was comparable among all groups in peel 1 (*P* = 0.597), whereas significant differences were observed in peel 2 (*P* = 0.034). Here, the lowest values were observed in the Co and Zo + Be groups (Fig. 6e and f). Wide quartile ranges of Tb.Sp values were seen in peel 1 for De + Be, Zo + Be, and Zo groups. In peel 2, low values were noted in all groups. The statistical analyses revealed no significant between-group differences for peel 1 (P=0.649) and peel 2 (*P* = 0.680) (Fig. 5g and h).

The BS/BV values were comparable among all groups in peel 1 (*P* = 0.671), whereas significant differences among groups were observed in peel 2 (*P* = 0.028). Here, the highest values were noted in the Co group (Fig. 6i and j).

## Discussion

The present study aimed at evaluating the influence of various antiresorptive and antiangiogenic medications on the extension of experimentally induced peri-implantitis lesions in an established rodent model [23, 24]. In fact, the experimental procedure was associated with the occurrence of clinical signs of peri-implant mucosal inflammation, a chronic-type ICT and a progressive loss of the implant-supporting bone. Basically, the histomorphometric analysis revealed comparable median aICT (lowest—Zo: 0.53 mm; highest—Be: 1.22 mm), defect length (lowest—Zo: 0.90 mm; highest—Co: 1.93 mm), and defect width (lowest—De + Be: 1.27 mm; highest—Be: 1.80 mm) values in all test and control groups investigated. A direct correlation of these histological outcomes with clinical signs of inflammation was not deemed feasible due to limitations in accurately assessing relevant outcome measures such as probing pocket depths or suppuration in this rodent model.

Moreover, it was noted that all test and control groups revealed comparable characteristics of the ICT, as indicated by the frequency and distribution of CD68-positive cells. Accordingly, these inflammatory cell infiltrates do share several similarities with those noted either at experimentally induced peri-implantitis lesions in the canine or naturally occurring peri-implantitis lesions in humans [20, 21, 31]. In this context, it must be realized that peri-implantitis lesions reveal a rather complex cellular composition (e.g. T cells, B cells, plasma cells) of the inflammatory infiltrate [20], thus requiring more detailed immunohistochemical analyses to elucidate potential differences between the various test and control groups investigated. The latter was however not the focus of the present study and the selection of CD68 just to confirm the inflammatory nature of the established ICTs in this animal model.

Previous preclinical and clinical data have pointed to the potential of Zo to induce pro-inflammatory effects, mainly affecting the polarization of M1 macrophages [12, 32], which are of particular relevance in the pathogenesis of peri-implantitis [33]. Infectious events have also been linked with the administration of Be and were mainly related to severe febrile neutropenia and the occurrence of fistulae/ abscesses in cancer patients [34]. Similar events could not be observed in the present study, as verified by the histological and immunohistochemical outcomes assessed.

When further analyzing the bone microstructures, it was noted that within both peel 1 and peel 2, there were no significant differences between the test and control groups investigated. In this context, it must be emphasized that this is the first analysis of established outcome measures to assess the bone microstructure [35–39] at peri-implantitis sites, particularly under antiresorptive/ antiangiogenic medications. Accordingly, any comparison with other findings is not feasible.

A recent preclinical study has focused on the analyses of periodontal and bone microstructures under Zo therapy [36]. In particular, at 10 months following a repeated drug administration (50μg/kg) in rabbits, Zo was associated with significantly higher periodontal space thickness (P.Th) values at non-infected maxillary premolars and 2nd molars when compared with the control (no Zo) group. While a trend for increased Tb.Th values in the Zo group was apparent, the analysis however did not reveal any significant between-group differences for mean BV/TV, BMD, Tb.Th, or BS/BV values [36].

The latter finding was basically in agreement with the results of two previous μCT analyses performed in rats [40, 41], indicating that Zo (66 μg/kg; 3× per week) was associated with higher mean Tb.Th values of 130.901 ± 22.850 μm and increased BV/TV values of 46.113 ± 8.56% measured in pristine areas, as opposed to 113.438 ± 28.131 μm and 34.180 ± 17.516% noted in the control animals (no Zo), respectively [40]. Increased Tb.Th and BV/TV values were also reported at the interradicular bone at the 1st molar as well as the periapical region adjacent to the 1st and 2nd molars at 21 weeks following Zo administration (0.6 mg/kg; 3× per month) [41]. A major limitation of the latter studies was however the lack of any induced periodontal/ periapical inflammatory lesions. While antiresorptive drugs basically tend to be associated with increased Tb.Th and BV/TV values, these effects may be neutralized by the pro-inflammatory environment and resulting progressive bone loss noted at, e.g., diseased implant sites. This was basically confirmed by the present analysis of both VOIs depicting either the inner or outer peels. The true effect of Be, Zo + Be, De, and De + Be on the bone micromophometry in general needs to be further investigated employing non-diseased implant sites.

Another major observation of the present study was the high frequency of implant losses noted during the progression period. Regrettably, the exact time points of the implant losses in various groups could not be determined. However, since these events were equally distributed among test and control groups, they may not be attributed to the administration of antiresorptive/antiangiogenic medications per se, but rather to the progressing bone loss at relatively short implants. In this context, it must be emphasized that Nguyen Vo et al. [27] employing even shorter machined implants in a similar mouse model did not report on any implant losses over a period of 4 weeks. This obvious difference noted in implant losses might be mainly attributed to the extended period of 16 weeks defined for the establishment of experimental peri-implantitis lesions in the present study. While the usage of rough-surfaced implants might have resulted in a lower frequency of implant losses, this model necessitated the application of a machined surface to facilitate implant removal during histological processing. In fact, the anatomical limitations associated with this experimental rodent model and subsequently the usage of ultra-narrow-diameter implants commonly necessitated a decalcified tissue sectioning [23, 24, 27], which in turn precludes the evaluation of the implant-bone interface.

When further evaluating the present analysis of sites exhibiting an implant loss, there was a trend noted towards lower defect width values in the Co group, thus potentially indicating that defect closure might have been impaired in the test groups. The latter was particularly true for Zo + Be, De, and Be groups, respectively. Nevertheless, histological signs of an osteonecrosis zone (i.e., reduced number of osteocytes and presence of empty lacunae) were commonly not observed in any of the specimens evaluated. Recent preclinical studies also reported on a compromised extraction socket healing under Zo therapy [42], particularly in the presence of experimentally induced periodontal infections [43].

In conclusion, the present analysis did not reveal any marked effects of various antiresorptive/antiangiogenic medications on the extension of experimentally induced peri-implantitis lesions.

## Data Availability

Data will be provided upon reasonable request.
